# A New Framework for Monitoring and Evaluating Health Impact Assessment: Capitalising on a French Case Study with the Literature in Evaluation

**DOI:** 10.3390/ijerph21091240

**Published:** 2024-09-19

**Authors:** Françoise Jabot, Julie Romagon, Guilhem Dardier

**Affiliations:** Univ Rennes, EHESP, CNRS, ARENES—UMR 6051, F-35000 Rennes, France; jromagon@gmail.com (J.R.); guilhem.dardier@ehesp.fr (G.D.)

**Keywords:** health impact assessment, effectiveness, theory-based evaluation, realist evaluation, logic model

## Abstract

Health impact assessment (HIA) is a prospective approach that aims to identify the potential consequences of policies or projects on health in order to propose measures to make them healthier. Initiated in the late nineties, the approach emerged over ten years ago in France. However, the evaluation of HIA effectiveness remains seldomly practised and its theoretical background should be deepened. The aim of this article is to generate a discussion on how to evaluate HIA effectiveness and contribute to its methodological tooling, drawing on an evaluative experience of multiple French HIAs. Our work is based on an iterative approach between an analysis of the evaluation literature and a critical look at an HIA evaluation. We first carried out the evaluation of three HIAs in 2017–2018, combining a normative approach and qualitative research in order to explore each HIA as a phenomenon in its own context. Two years later, we conducted a self-assessing expertise on this evaluation, supported by an analysis of the literature in the field of public policy evaluation, in order to refine the theoretical framework for evaluating HIA effectiveness and ultimately to enhance professional practice by evaluators. This work led to the production of a logic model that identifies, through three dimensions (context, implementation and governance), the multiple pathways that HIA may take to bring about change. It also seeks to show the interdependence of these pathways towards change and helps identify the key drivers and mechanisms of HIA success. In this respect, it complements existing HIA evaluation models as it can serve both as a generic framework for evaluating HIA effectiveness and as an instrument for monitoring HIA implementation.

## 1. Introduction

Health Impact Assessment (HIA) aims to prospectively assess the potential consequences of a policy, programme, or plan on the health of a population in order to propose measures to mitigate potential negative impacts and reinforce positive impacts. Its purpose is to ensure policies are more favourable to health [1,2]. HIA emerged in the late nineties [3] and expanded internationally [4,5]. The international community has adopted an HIA methodology and ethics framework. HIA follows a structured process in six steps: determine whether or not HIA is relevant and feasible (screening), set out the scope and the parameters of HIA (scoping), collect, analyse data, and assess health impacts (assessment), elaborate a set of recommendations (reporting), set up mechanisms for the follow-up of recommendations (monitoring), evaluate process and outcomes of HIA (evaluation). Various guides have been produced covering best practices, objectives, stages, and tasks [6]. However, diversity in HIA practice exists due to its flexibility of focus, contexts, goals, and methods [4,7]. Nevertheless, as recalled in a recent article [8], since “HIAs are intended to support decision-making and implementation prior to an action”, the term should not be applied to the studies—like some impact modelling exercises and quantitative health risk assessments—that might assess health impacts but are “unlikely to influence current decision-making” on a specific intervention. 

The HIA process includes evaluation, which covers three strands. Quality analysis ensures HIA complies with practice standards since quality data production is considered an essential factor of credibility and effectiveness [9,10,11]. The process evaluation also looks into governance, stakeholder engagement, available resources, communication, and how HIA has been tailored to the context. The evaluation of outcomes refers to the ability of HIA to inform policymaking and to support the advancement of equity, health, and well-being in plans and policies [12]. However, HIA evaluation remains seldom practiced and knowledge about its effects remains insufficient [13]. Evaluating the effectiveness of HIA is not an easy task, and the very idea of the effectiveness of impact assessment needs to be clarified since there is a diversity of perspectives [14]. Several authors have thus developed theoretical frameworks or methods for evaluating HIA effectiveness [12,15,16,17,18,19,20], and the research should, therefore, be shared and discussed. 

In France, HIA emerged just over ten years ago and has grown significantly in the last few years [21] (Jabot, 2021). It has mainly developed at the local level, driven by some regional health agencies (RHA). They have been keen to lobby local authorities for the use of the approach in their town planning. Progressively, French municipalities and RHAs have commissioned HIAs, being aware that this approach is a promising way of integrating health into other policies, particularly through more targeted action on social inequalities in health. Three RHAs have developed specific strategies for deploying the HIA approach and have undertaken evaluations in order to decide whether to continue and/or extend this new policy. 

The RHA for the Pays de la Loire region (RHA-PdL) has taken an interest in HIA since 2013, particularly on urban development policies and projects, and funded in 2016 three HIAs [21] (Jabot, 2021). A year later, the agency evaluated how these three HIAs had been implemented and what their benefits were. The aim of this evaluation was to set out the conditions for a successful wider HIA deployment. This was the first evaluation of HIA in France, and it drew the attention of other regional stakeholders who sought to introduce or continue the approach themselves. The RHA for the Ile-de-France region (RHA-IdF) evaluated its policy dedicated to the development of healthy urban planning through HIA or urban development project support a few years later, and the RHA for the Nouvelle Aquitaine region (RHA-NA) just completed the evaluation of its specific organisation that has enabled the implementation of some twenty HIAs. 

Decision-makers in France are increasingly showing strong interest in HIA and are awaiting evidence for its added value over other approaches (exploratory walks, citizen engagement, project support, etc.). This observation supports the need for conducting reliable evaluations to ensure HIA is credible and continues to be used [22] (Jabot et al., 2022). The aim of this article is to generate further discussion on how to evaluate HIA effectiveness.

Following a review of the literature on effectiveness evaluation, particularly in the field of HIA, and building on our experience in HIA evaluation, we will present a new theoretical framework with a supportive tool (a generic HIA logic model) that can be used for future HIA evaluations. Finally, we will discuss the benefits and limitations of this new logic model. 

## 2. Material and Methods

Our work is based on an iterative approach between an analysis of the evaluation literature and a critical look at an HIA evaluation. We followed three stages. 

### 2.1. Carrying Out the HIA PdL Evaluation

First, we carried out, in 2017–2018, the evaluation of the three HIAs mandated by the RHA-PdL. This evaluation attempted to answer questions related to the quality of HIAs implementation and their influence in terms of health and the reduction in health inequalities, to the key factors explaining the success or failure of an HIA, and to the best conditions to deploy HIAs in the region, with specific recommendations regarding the role of the Regional Health Agency. Our evaluation strategy drew on a comparative case method [23], with each HIA taken as a case and studied with the scope of the evaluation questions. We combined a normative approach along with a set of standards and qualitative research to evaluate the outcomes as a change in the project examined, as well as a change in the representations and practices of stakeholders and institutions. Drawing on previous work in evaluating evaluation [24] and HIA effectiveness [17,25,26], we produced a table with the different types of change, identifiable at different levels (individual, interpersonal, and collective), with their different natures (proximal, distal) and short-, medium-, and long-term timescales (Table 1). 

We chose with the technical committee six success criteria: capacity to adjust the project, capacity to make stakeholders work together, capacity to develop a culture of health among decision-makers, capacity to change professional practices, capacity to foster citizen involvement and participation, and capacity to make HIA recognised and understood by stakeholders and institutions. The information collected was analysed within this framework in order to identify which factors had a perceptible influence on the success or failure of HIA, the prerequisites for HIA implementation, and on which institutions may be able to act. 

The success factors we identified were put into three categories related to: the quality of the HIA method (alignment with the standards, methodological tools, use of evidence, mixed approaches, close relation evaluator-institutional liaison officer, external methodological support); the implementation process (early awareness by the services concerned, participation of a large panel of stakeholders, work within mixed groups, co-construction of recommendations, involvement of the recipients of the recommendations); the political will and institutional strategies deployed in favour of HIA (inclusion of HIA in contractual documents, policy or planning instruments, identification of a dedicated department or team, provision of financial resources, mobilisation of partners, anticipation of follow-up). The final report was delivered in early 2018 with useful insights for HIA practice and highlighting certain changes in terms of representations of health among elected representatives, as well as the ways in which institutions work together. However, the evaluation timescale did not allow the changes in the projects themselves to be observed. 

At the time, we would have liked to go deeper into the HIA method, but the timeline of evaluation had to be aligned with the decision-making agenda as the RHA-PdL wanted to use the findings to push ahead with its strategy. These factors are well-known limits of the real-world evaluation [27]. Thus, two years after the PdL evaluation, we conducted a self-assessing expertise [28] of the work in order to refine the theoretical framework for evaluating HIA effectiveness, to improve our practice and ultimately to enhance professional practice by evaluators.

### 2.2. Literature Analysis

To do so, we first analysed literature in the field of public policy evaluation to clarify the concept of effectiveness of evaluation (what does it mean? Why and how do we consider an evaluation as effective?), to review the methods to assess the effectiveness and to identify the way in which this issue is addressed in the field of HIA. Thus, we examined, in the light of evaluation research, the most recent existing frameworks for assessing the effectiveness of HIA and their ability to account for the effectiveness.

### 2.3. Reflexive Path to a Theoretical Framework

Our main questions were: Did the HIA evaluation design really/truly meet its commissioners’ expectations? Was this evaluation useful? How could we have done better? We were aware of the limitations of the evaluation. Particularly, more time and remote working would have been needed in order to explore the mechanisms with more depth and to analyse the change process set against the wider interactions between the context, players, and HIA, in line with the theory-based evaluation approach that underpins our practice. 

Then, after gaining perspective and insights into the scientific literature, we went back to the initial theoretical framework of the HIA PdL Evaluation and reconstructed a change theory. The theory was implicit but had not been sufficiently explained when the evaluation took place, although it was consolidated later using the conclusions of the evaluation. We also examined the two other regional HIA evaluations in which we were involved to various degrees to support our understanding of the context in which HIAs are implemented. In the end, we were able to produce a new theoretical framework with a supportive tool (the generic HIA model) that can be used for future HIA evaluations. 

## 3. Results: Towards a New Theoretical Framework for HIA Monitoring and Evaluation

### 3.1. The Literature on Effectiveness in and of Evaluation

Evaluation is a specific activity that consists of making a judgement about an intervention in the light of evaluative questions with different aims: to clarify and support the decision, to report on what has been conducted, or to improve the intervention concerned by the evaluation [29,30]. Evaluation is considered to be effective if it achieves the goal(s) assigned to it. However, evaluation is not just a methodological exercise (assessment); it is a process embedded in a system of dynamic relationships between stakeholders, institutions, practices, a context, and its own timeframe (evaluation) [31]. In this section, we will clarify what is meant by the “effectiveness of evaluation,” i.e., its ability to bring change (Section 3.1.1), discuss the ways in which“ evaluation of effectiveness” is addressed as a process because of its interactions with the environment in which it takes place and its potential for change (Section 3.1.2), and their application in the context of HIA, which is a particular form of evaluation (Section 3.1.3).

#### 3.1.1. Concept of Effectiveness: Between Use and Influence

Questions about the effectiveness of evaluation have concerned researchers for decades [32] and have been widely explored [33,34,35,36,37,38]. The effectiveness of evaluation is considered through its usefulness, i.e., whether it affects policy decisions and produces relevant knowledge to the policy being evaluated, provides a fresh understanding of the reasons for its success, and puts forward new hypotheses about a particular intervention. The concept of use has been explored, the main factors at play have been identified, and issues specific to the link between research and action have been brought to light. There are multiple forms of evaluation used. To draw on the conclusions and recommendations of an evaluation report (instrumental use) is only one form of use. Another is conceptual enlightenment, which informs decision-making by reframing representations, concept maps, and even practices by individuals and organisations [38]. Evaluation can also be used to support and legitimise positions (symbolic use). Kirkhart broadened the understanding of evaluation use in terms of influence, defined as “[the] capacity or power of individuals and things to produce effects on others by non-tangible or indirect means” [39]. This author analysed influence according to source (actors, change process), intention, and time in which the mechanisms operate. Henry and Mark [40] drew on her work and on programme theory to develop a logic model for appraising the impacts of evaluation. Although evaluation use means intention and awareness and it comes from the power of decision-makers, influence itself is indirect and takes multiple pathways [40]. The theory of influence links evaluation activities to short-, medium-, and long-term outcomes, whether direct and easily objectified or indirect and more difficult to track. Their proposed model examines the effects and mechanisms of change on three levels: individual, interpersonal, and collective. It thereby links use types and mechanisms: conceptual use and cognitive and emotional processes; instrumental use and change process; symbolic use and interpersonal and collective processes; processual use and activities [40].

#### 3.1.2. A Range of Approaches to Evaluate Effectiveness

Evaluation of effectiveness (occurrence of outcome as changes) has to demonstrate the difference between what happened after the intervention and what would have happened without it. This is the counterfactual principle. In this respect, several approaches coexist, some using experimental methods (based on measure and comparison between two situations), others “based on the theory”, striving to explain what happens between an intervention and its effects, what works, and why. As Weiss [41] stated, “a programme is a theory, and the evaluation is its test; the evaluator needs to understand the theoretical premises on which the program is based”. Thus, theory-based evaluation (TBE) is an explicit theory of change aiming to draw conclusions about whether and how an intervention would produce expected results [42]. The evaluation seeks to test the theory elaborated, and diverse methods/approaches have been developed. Realist evaluation [43] looks at the interactions between the mechanisms at work in an intervention, the context, and the outcomes. Contribution analysis seeks to make credible causal claims from a set of hypotheses, known as supporting factors, for change to take place, and it discards alternative explanations [44]. In the same vein, the process tracing method attempts to unpack causal processes by detailing what happens in a programme at each “key episode” to understand its contribution to change [45]. A key element of TBE is the analysis of the causal mechanisms because it shows the relevance of evaluation [46]. The founders of realist evaluation, Pawson and Tilley [43], postulate that outcomes are produced by mechanisms in a context: (C + M→O). Although introduced more than twenty years ago, the concept of mechanism is still difficult to define. A mechanism is considered a component of the programme, a response to the programme, or an explanatory element [47]. The difficulty in distinguishing context and mechanism means that the configurations should be reviewed [47]. Punton and Vogel [48] developed the CIMO configuration: an intervention interacting with its context triggers a mechanism and produces an outcome (C + I→M→O). 

Reichardt [49], looking at seven common approaches to assessing effects, concluded that TBE can be used to show the difference as it reports the links between the outcomes and the interventions. In this way, it demonstrates “counterfactual thinking” and can be considered a valid approach for the evaluation of complex interventions.

#### 3.1.3. Evaluating HIA Effectiveness

HIA is relatively recent with respect to “classical” evaluation, and the question of evaluating its effectiveness as an activity in its own right has been less thoroughly explored. Authors have studied the concept of effectiveness in the field of impact assessments [14]. Most studies on HIA’s effectiveness have focused on HIA use and success factors [15,16,25,26,50,51,52,53]. Only a few have studied the impact of its recommendations [54] or considered HIA as an intervention [55].

As for “classic evaluation”, evaluating HIA effectiveness involves addressing well-identified methodological challenges [55]: clarifying the nature of the outcome of interest (better health of the population or public policy that is more favourable to health?), assessing the feasibility of the evaluation, and constructing a suitable theoretical framework. Many questions have to be answered: is the gain objectively measurable, at what level (determinant or overall health), and at what time? Does one examine intermediate stages, i.e., effects on particular targets, and/or what explains (mechanisms) the change? What is the proportion of certain project components? For future HIA evaluations, it will be essential to clearly determine the needs, formulate evaluation questions in line with the HIA timescale and future uses, and establish appropriate terms of reference. 

Some authors have proposed a three-column theoretical framework (context, process, impacts) with descriptive variables for each [19,20,25,56]. That of Haigh et al. [25] includes cross-cutting factors in order to distinguish between different levels of effectiveness. These frameworks are structured in “Concept/Process/Impacts” and differ from logic models, which show causal chains and take into account interactions between levels of outcome. Therefore, they are not completely compatible with the TBE approach. Nour et al. [18] went a step further by drawing on contribution analysis to evaluate the effects of HIA, an approach deriving from theory-based evaluation. They showed the feasibility and utility of this approach to assess HIA processes and called for further work to consolidate this method of assessing the effects of HIA. 

In light of these studies, we wanted to take the theory a step further, look more closely at the levers of change, sometimes referred to as influencing factors or mechanisms, and provide a practical tool for evaluators.

### 3.2. Building a New Logic Model

TBE provides a conceptual framework for documenting why and how a programme produces change. The graphic representation of this theory in a diagram is known as the logic model. Its structure as “Intervention/Outputs/Intermediate and Long-term Outcomes” makes it possible to establish causal chains, identify mechanisms that operate to produce effects, and estimate interactions between these effects. 

#### 3.2.1. HIA as a Complex Intervention

First, we considered HIA an intervention in its own right and, more specifically, as a complex intervention for various reasons. It covers a set of activities with a range of participants with varying levels of involvement, different approaches and expectations, and who do not always share the same frames of reference [57,58,59]. HIAs are conducted in a variety of settings, institutional, political, and social, shaping the process. They are also complex due to alternative causal strands, the interactions between the different components, and the uncertainty of outcomes, being dependent on the unpredictable contribution of stakeholders and subject to unforeseen contextual circumstances. Therefore, they have non-linear effects due to some feedback loops. For example, the initial political context of the proposal strongly affects HIA implementation and its outputs, but launching and carrying the HIA can also change the political nature of the proposal, thus influencing its own implementation process and its effects. Given this complex nature, an adapted and dynamic approach is required for this type of intervention: identify what causes change and take into account the interactions between HIA and its context with respect to the constraints related to divergences, uncertainty, and timescale [59,60].

We broke down HIA into three components: HIA activity (implementation of the method), the setting in which it takes place (policy and administrative context), and the arrangement between actors of its implementation (governance). 

#### 3.2.2. Causal Chains, Mechanisms and/or Success Factors

Secondly, we established the causal chains of the changes observed that show the cascade of effects that HIA can generate at various levels (personal, collective, institutional) by explaining the conditions of these changes (success factors/mechanisms), all in a dynamic perspective that takes into account the interactions between these effects. We adopted the CIMO configuration (C + I→M→O) suggested by Punton et Vogel [48] as this configuration matched the conception of HIA as an intervention that brings together evaluation (as assessment activity), actors, and context all as a whole.

To build the model, we had to determine what change is (a shift from one state to another), what a mechanism is (that which produces/triggers a change), and what we had previously identified as success factors (factors that help produce change). Since we were unable to disentangle the two notions, we considered “success factor” and “mechanism” to be the same notion of causal mechanism defined as “pathways, processes, and intervening variables that causally link interventions to changes in outcome” [46].

For the mechanisms, we took the success factors identified during the PdL evaluation and compared them with the highly detailed list by Mark and Henry [24], keeping only those factors that were relevant to our study. These authors identified mechanisms at three levels: individual, “when evaluation processes or findings directly cause some change in the thoughts or actions of one or more individuals”; interpersonal, “a change brought about in interactions between individuals”; and collective, referring to “the direct or indirect influence of evaluation on the decisions and practices of organisations”. We agreed with this categorisation and adopted mechanisms (salience, justification, dissemination, collective learning, persuasion) that we observed as triggers or mediators between output and outcome or between outcomes. For example, “priming” is a mechanism because participation in HIA raises awareness of non-health sectors. We also attempted to differentiate mechanisms from outcomes. As opposed to Mark and Henry, we considered “behavioural change” and “policy change” more as outcomes than as mechanisms precisely because they are changes. We added mechanisms that were not on their lists, such as prioritisation, social reward, alliance building, readability, and ritualism. Furthermore, we attempted to differentiate mechanisms from the influencing factors. For example, we agreed that “time” and “timeliness” are factors that influence effectiveness [25], but they cannot be considered as mechanisms that produce change.

This initial outline was revised in light of the work of several authors [16,18,24,25] by adding, revising, or eliminating certain mechanisms and thus producing a version of a theoretical framework for evaluating HIA effectiveness.

#### 3.2.3. The HIA Logic Model

**Figure 1:**
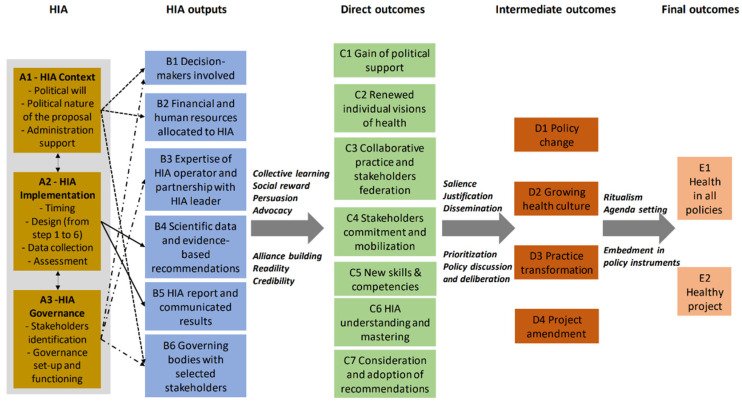
Logic model: the theory of HIA influence.

From the HIA intervention (column A) and its achievements (column B), the logic model (Figure 1) traces the pathways used to generate a series of changes/outcomes (direct/column C and intermediate/column D) leading to more health in projects and policy (column E). The arrows between columns B-C, C-D, and D-E represent the mechanisms.

HIA (column A) is divided into three components: (A1) the context (conflictual or unifying nature of the project studied, political will, support by the political administration, administrative framework); (A2) the evaluation activity (methodological framework, data collection, impact assessment, timescale); (A3) governance (stakeholder presence and participation, functioning). The context of the HIA (A1) influences the involvement of policymakers (B1), the allocation of financial and human resources to the process (B2), and the composition and functioning of decision-making bodies (B6). The evaluation activity (A2), in compliance with quality standards, leads to the production of scientifically constructed knowledge and evidence-based recommendations (B4) that are shared and set out in a report (B5). The governance of the HIA (A3) determines the choice of stakeholders, the nature of their involvement and the functioning of decision-making bodies (B6), the expertise sought through the evaluator, and the nature of the partnership with the institution that commissioned the HIA (B3). 

When HIA is conducted in this way and according to mechanisms of collective learning and social reward, the intervention may or may not foster the following: the full involvement of actors (C3–C4), their understanding of HIA, and their adoption of the concepts and instruments used (C6). These elements are necessary for upskilling (C5), for changes in individual and collective representations about health (C2), and, indirectly, for the creation of a common culture (D2) and a change in practices (D3).

The process of producing the HIA recommendations (co-construction, scientific substantiation, transparency) encourages actors—through mechanisms of visibility, readability, and credibility—to accept, consider, and implement them in the project for which HIA takes place (C7 and D4). Through advocacy and alliance-building mechanisms, federations of actors (C3) may or may not be formed, and political support may or may not be gained (C1), influencing the degree of consideration given to HIA (salience) when weighing its conclusions against other considerations (prioritisation). HIA is then more likely to generate policy change (D1) as a result of policy discussion and deliberation, whether the change is cognitive or operational. This change then becomes more easily actionable and replicable, thanks to it becoming embedded in routine practices (ritualism) and public policy instruments (inclusion in policy instruments), thus contributing to the integration of health in all policies (E1) and the development of projects that promote health (E2). 

## 4. Discussion

### 4.1. Benefits of the Model 

Theory-based evaluation (TBE) is a relevant approach due to its capacity to question the rationale of an intervention to highlight the interactions between the latter, the context, and the intermediate outcome. The logic model is the visual part of the theory of change, and as a tool, it has some benefits. It facilitates a common understanding of what the intervention is and should produce. It provides a theoretical framework for selecting the most suitable type of evaluation, the issues to explore, and the changes to report. It also serves to identify the information to collect and that is available and to explore those factors that may influence the intervention.

When the logic model is applied to HIA, it enables the key points of the evaluation to be addressed in line with methodological goals: define the nature of the outcome to be evaluated, analyse its evaluability, set the scope of the evaluation (implementation and effects of recommendations, changes in practices, new skills, integration of health into other policies, etc.). It also helps identify the mechanisms at the different levels (individual, processual, institutional) to understand how HIA is an effective strategy for change, namely, including health in all policies (HiAP).

When the logic model is discussed with decision-makers, it should then offer a systemic vision of HIA and facilitate the assessment’s evaluability and acceptability. The mechanisms should be transparent so that stakeholders can understand what the conditions for success are [46]. The mechanisms at work on the individual level influence the choices, perceptions, representations, and behaviours of those involved. They are mainly related to actors’ responses and are combined with process mechanisms, defined as a “sequence of interactions in which actors engage in activities, transmitting causal forces from intervention to outcome” [46]. Together, they trigger dynamics (between columns B and C) at the interpersonal level (advocacy, collective learning, alliance building, social reward) [48]. The mechanisms between columns C, D, and E (policy deliberation, ritualism, agenda setting, etc.), which are responsible for more distal effects, i.e., administrative and political formalisation, belong to the institutional level. This logic model can also be useful for monitoring the HIA to ensure that the conditions for its success are in place. Used at the screening stage, where the decision to launch the HIA is made, it will allow the following elements to be examined: favourable governance, right timing, political and administrative support, necessary resources, and availability of information. This logic model provides useful guidance for HIA during the implementation of each stage (quality of the method, production of data, commitment of stakeholders, effective participation, and regular communication).

As mentioned before, our work builds on the existing literature on evaluation and HIA effectiveness. The Mark and Henry model [24] was very influential in that they view evaluation as an intervention with a reconstructed logic of action and because of their in-depth reflection on mechanisms. Nevertheless, it needed to be presented in a more dynamic way and adapted to HIA with revisions on mechanisms. Harris–Roxas and Harris conceptual framework [17], revised by Haigh et al. [25], was in line with previous work [19,20] and provides a strong framework for the success factors identified in the literature and in studies conducted in Australia and New Zealand, but it is not compatible with TBE. Therefore, our model differs from the existing ones in several aspects. Firstly, our logic model is dynamic in nature. HIA is considered as an intervention divided into three components that produce direct (or proximal) and then indirect (or distal) effects. It is not a static framework since it explores the operating mechanisms, considers parallel and/or alternative causal pathways, and analyses the interactions between different effects at different levels (individuals, institutions, and organisations). In doing so, it is in line with the process theory of change approach, which considers that “real-world project interventions are typically not activities performed at a particular moment in time but instead are a series of interactions with other actors (…) over a period of time during which a program is implemented“ [45]. Secondly, it was built with reference to theoretical approaches in evaluation. It is based on theory-based evaluation and its derived concepts and takes into account the complexity dimension [57,59]. Thirdly, it has several applications and can be used both for the evaluation of HIA and during its implementation. During the HIA evaluation, it is used to assess effectiveness through the analysis of causal chains and exploration of mechanisms, but also at the beginning of the evaluation to choose evaluative questions, identify data to be collected, and estimate evaluability. During the HIA process, it allows to ensure the conditions for the success of HIA are in place, particularly at the time of screening but also during the course of HIA.

### 4.2. Limitations of the Model

The work presented does incur some limitations. Firstly, the distinction between component, outcome, and mechanism remains difficult to make, given the plural nature of mechanisms. A mechanism in one context may well be considered a component of HIA in another context [47]. For example, advocacy, a concept we prefer to persuasion because it is more in line with the values of health promotion, is a deliberate strategy for HIA and constitutes, both in France and in Quebec, a step prior to screening to promote the approach to municipalities [61] (Jabot et al., 2020). However, advocacy remains a lever to be activated in the entire process. In the same way, the process of adoption presented here as a necessary mechanism for integrating the recommendations and systematising the approach can be seen as a result of participation in HIA.

Secondly, the development of this model, although partly based on the literature and informed by our experience in the HIA field, only draws on the results from an evaluation of three HIAs, which limits the variety of objects concerned in the French political, institutional, and organisational context; indeed, certain changes and mechanisms are likely to be expressed differently in other contexts and on other objects. It should be tested in other HIAs with more hindsight on the effects but also more broadly on the consideration of health in all policies.

### 4.3. Avenues for Future Research

Evaluation of HIA effectiveness continues to develop with research showing methodological innovations. In recent years, some authors [18,62] have drawn on contribution analysis, which is a promising method and completes realist evaluation. On the one hand, it provides a practical framework with a sequenced, multi-step method for testing all hypotheses whereby the intervention may play a role in producing change by revealing supporting factors. On the other hand, it eliminates rival explanations unrelated to the intervention [44,47].

We focused on realist evaluation because it was possible to reconstruct the theory of change a posteriori, as the ingredients were present in the evaluation carried out in the PdL region, and this initial work on mechanisms was useful for understanding causality and developing a generic model for evaluating the HIA approach (and not a specific HIA). Furthermore, what induces change keeps questioning researchers: a mechanism for realist evaluation, supporting factor for contribution analysis, or stimulus, which, according to Alkin and King [38], “leads to potential use”. There is room for further discussion, room to question the conceptual distinction between mechanism and supporting factor and stimulus, and, if it exists, its consequences for evaluation. TBE methods and their variants, such as process tracing, need to be further experimented to better understand whether, why, and how HIAs are a means of changing non-health-related public policies and, in so doing, improving the well-being and health of people.

## 5. Conclusions

The logic model presented in this article seeks to identify, through three dimensions (context, implementation, and governance), the multiple pathways that HIA may take to bring about change. It also seeks to show the interdependence of these pathways towards change and helps to identify the key drivers of HIA success. In this respect, our model complements existing HIA evaluation models [14,16,17], as it can serve both as a generic tool for evaluating HIA effectiveness and as an instrument for monitoring HIA implementation. It is not relevant to assess the health impact on the population, which may be affected by multiple other causes [15].

Today, decision-makers are concerned with results in terms of health indicators, the reduction in inequalities, or monetary added value. They want to know if the action carried out produces changes and why so that it can be replicated elsewhere or with other populations. One way of aligning decision-makers’ expectations with the evaluation’s capabilities would be to use a logic model that explains the hypothesis of changes, i.e., the paths leading to them and the conditions for achieving them. Given its characteristics (opening the black box, attributing failures to theory or implementation, taking complexity into account), theory-based evaluation is a relevant approach because it provides knowledge, thus ushering in the 5th generation of evaluation, “the explanation generation” [63]. If the answers expected are not found, then this may hinder the more systematic integration of HIA into decision-making processes. It is, therefore, crucial to co-develop robust evaluations by involving decision-makers [64].

## Figures and Tables

**Table 1:** Types of changes.

Project-related changes Project amendment as a result of the implementation of the recommendations New decisions based on HIA Integration of HIA findings in policy instruments and other policies
Institution-related changes Evolution of the ways of intervention Evolution of practices Evolution of relations between actors who participated in the HIA Closer or new partnerships
Individual-related changes Evolution of stakeholders’ health and equity representations [all stakeholders] Growing awareness of the consequences of decisions on health determinants [decision-makers] Capacity building (skills, power…) [technicians, citizens] Appropriation of the HIA approach [technicians]
Policy-related changes Better integration of health in all policies Addressing the roots of social inequalities in health Solutions to reduce or offset/counterbalance inequalities

## Data Availability

The data presented in this study are available on request from the corresponding author. The data are not publicly available due to privacy restrictions.

## References

[1] Kemm J. (2001). Health Impact Assessment: A tool for Healthy Public Policy. Health Promot. Int..

[2] Winkler M.S., Viliani F., Knoblauch A.M., Cave B., Divall M., Ramesh G., Furu P. (2021). Health Impact Assessment International Best Practice Principles.

[3] World Health Organization (1999). Health Impact Assessment: Main Concepts and Suggested Approach, Gothenburg Consensus Paper, Bruxelles.

[4] Winkler M.S., Furu P., Viliani F., Cave B., Divall M., Ramesh G., Harris-Roxas B., Knoblauch A.M. (2020). Current Global Health Impact Assessment Practice. Int. J. Environ. Res. Public Health.

[5] Harris-Roxas B., Viliani F., Bond A., Cave B., Divall M., Furu P., Harris P., Soeberg M., Wernham A., Winkler M. (2012). Health impact assessment: The state of the art. Impact Assess. Proj. Apprais..

[6] Hebert K.A., Wendel A.M., Kennedy S.K., Dannenberg A.L. (2012). Health impact assessment: A comparison of 45 local, national, and international guidelines. Environ. Impact Assess. Rev..

[7] Harris-Roxas B., Harris E. (2011). Differing forms, differing purposes: A typology of health impact assessment. Environ. Impact Assess. Rev..

[8] Kim J., Dannenberg A., Haigh F., Harris-Roxas B. (2024). Let’s Be Clear—Health Impact Assessments or Assessing Health Impacts?. Public Health Rev..

[9] Bhatia R., Farhang L., Heller J., Lee M., Orenstein M., Richerdson M., Wernham A. (2014). Minimum Elements and Practice Standards for Health Impact Assessment.

[10] Fredsgaard M.W., Cave B., Bond A.J. (2009). A Review Package for Health Impact Assessment Reports of Development Projects.

[11] Green L., Parry-Williams L., Edmonds N. (2017). Quality Assurance Review Framework for Health Impact Assessment (HIA).

[12] Sohn E.K., Stein L.J., Wolpoff A., Lindberg R., Baum A., Simoncelli A.M.I., Pollack K. (2018). Avenues of Influence: The Relationship between Health Impact Assessment and Determinants of Health and Health Equity. J. Urban Health.

[13] Morteruel M., Bacigalupe A., Aldasoro E., Larrañaga I., Serrano E. (2020). Health Impact Assessments in Spain: Have They Been Effective?. Int. J. Environ. Res. Public. Health.

[14] Chanchitpricha C., Bond A. (2013). Conceptualising the effectiveness of impact assessment processes. Environ. Impact Assess. Rev..

[15] Ali S., O’Callaghan V., Middleton J.D., Little R. (2009). The challenges of evaluating a health impact assessment. Crit. Public Health.

[16] Haigh F., Baum F., Dannenberg A.L., Harris M.F., Harris-Roxas B., Keleher H., Kemp L., Morgan R., Chok H.N., Spickett J. (2013). The effectiveness of health impact assessment in influencing decision-making in Australia and New Zealand 2005–2009. BMC Public Health.

[17] Harris-Roxas B., Harris E. (2013). The impact and effectiveness of health impact assessment: A conceptual framework. Environ. Impact Assess. Rev..

[18] Nour K., Dutilly-Simard S., Brousselle A., Smits P., Buregeya J.-M., Loslier J., Denis J.-L. (2016). Evaluation of the effects of health impact assessment practice at the local level in Monteregie. Health Res. Policy Syst..

[19] Quigley R.J., Taylor L.C. (2004). Evaluating health impact assessment. Public Health.

[20] Wismar M., Blau J., Ernst K., Figueras J. (2007). The Effectiveness of Health Impact Assessment: Scope and Limitations of Supporting Decision-Making in Europe.

[21] Jabot F. (2021). L’évaluation d’impact sur la santé pour scruter et sculpter les politiques. Sante Publique.

[22] Jabot F., Rivadeneyra-Sicilia A. (2022). Health impact assessment institutionalisation in France: State of the art, challenges and perspectives. Impact Assess. Proj. Apprais..

[23] Yin R.K. (2009). Case Study Research: Design and Methods.

[24] Mark M.M., Henry G.T. (2004). The Mechanisms and Outcomes of Evaluation Influence. Evaluation.

[25] Haigh F., Harris E., Harris-Roxas B., Baum F., Dannenberg A.L., Harris M.F., Keleher H., Kemp L., Morgan R., Ng Chok H. (2015). What makes health impact assessments successful? Factors contributing to effectiveness in Australia and New Zealand. BMC Public Health.

[26] Dannenberg A.L. (2016). Effectiveness of Health Impact Assessments: A Synthesis of Data From Five Impact Evaluation Reports. Prev. Chronic. Dis..

[27] Bamberger M., Mabry L. (2019). RealWorld Evaluation: Working Under Budget, Time, Data, and Political Constraints.

[28] Stevahn L., Berger D.E., Tucker S.A., Rodell A. (2020). Using the 2018 AEA Evaluator Competencies for Effective Program Evaluation Practice. New Dir. Eval..

[29] Mathison S. (2005). Encyclopedia of Evaluation.

[30] Patton M.Q. (1997). Utilization-Focused Evaluation: The New Century Text.

[31] Rog D.J. (2012). When background becomes foreground: Toward context-sensitive evaluation practice. New Dir. Eval..

[32] Alkin M.C., King J.A. (2016). The Historical Development of Evaluation Use. Am. J. Eval..

[33] Johnson K., Greenseid L.O., Toal S.A., King J.A., Lawrenz F., Volkov B. (2009). Research on Evaluation Use A Review of the Empirical Literature from 1986 to 2005. Am. J. Eval..

[34] King J.A., Alkin M.C. (2019). The Centrality of Use: Theories of Evaluation Use and Influence and Thoughts on the First 50 Years of Use Research. Am. J. Eval..

[35] Leviton L.C., Hughes E.F. (1981). Research on the Utilization of Evaluations A Review and Synthesis. Eval. Rev..

[36] Shulha L.M., Cousins J.B. (1997). Evaluation Use: Theory, Research, and Practice Since 1986. Am. J. Eval..

[37] Weiss C.H. (1998). Have We Learned Anything New About the Use of Evaluation?. Am. J. Eval..

[38] Alkin M.C., King J.A. (2017). Definitions of Evaluation Use and Misuse, Evaluation Influence, and Factors Affecting Use. Am. J. Eval..

[39] Kirkhart K.E. (2000). Reconceptualizing evaluation use: An integrated theory of influence. New Dir. Eval..

[40] Henry G.T., Mark M.M. (2003). Beyond Use: Understanding Evaluation’s Influence on Attitudes and Actions. Am. J. Eval..

[41] Weiss C.H. (1997). Theory-based evaluation: Past, present, and future. New Dir. Eval..

[42] Funnell S.C., Rogers P.J. (2011). Purposeful Program Theory: Effective Use of Theories of Change and Logic Models.

[43] Pawson R., Tilley N. (1997). Realistic Evaluation.

[44] Mayne J. (2012). Contribution analysis: Coming of age?. Evaluation.

[45] Camacho Garland G., Beach D. (2023). Theorizing how interventions work in evaluation: Process-tracing methods and theorizing process theories of change. Evaluation.

[46] Schmitt J. (2020). The Causal Mechanism Claim in Evaluation: Does the Prophecy Fulfill?. New Dir. Eval..

[47] Lemire S., Kwako A., Nielsen S.B., Christie C.A., Donaldson S.I., Leeuw F.L. (2020). What Is This Thing Called a Mechanism? Findings From a Review of Realist Evaluations. New Dir. Eval..

[48] Punton M., Vogel I. (2020). Keeping it Real: Using Mechanisms to Promote Use in the Realist Evaluation of the Building Capacity to Use Research Evidence Program. New Dir. Eval..

[49] Reichardt C.S. (2022). The Counterfactual Definition of a Program Effect. Am. J. Eval..

[50] Gamache S., Diallo T., Lebel A. (2022). The use of health impact assessments performed in Quebec City (Canada)—2013–2019: Stakeholders and participants’ appreciation. Environ. Impact Assess. Rev..

[51] Davenport C., Mathers J., Parry J. (2006). Use of health impact assessment in incorporating health considerations in decision making. J. Epidemiol. Community Health.

[52] Cunningham D.R., Signal L., Bowers S., Wellington N.Z. (2011). Evaluating Health Impact Assessments in New Zealand.

[53] Bourcier E., Charbonneau D., Cahill C., Dannenberg A.L. (2015). An Evaluation of Health Impact Assessments in the United States, 2011–2014. Prev. Chronic. Dis..

[54] Mathias K.R., Harris-Roxas B. (2009). Process and impact evaluation of the Greater Christchurch Urban Development Strategy Health Impact Assessment. BMC Public Health.

[55] Buregeya J.M., Brousselle A., Nour K., Loignon C. (2017). Comment évaluer les effets des évaluations d’impact sur la santé: Le potentiel de l’analyse de contribution. Can. J. Program Eval..

[56] Parry J.M., Kemm J.R. (2005). Criteria for use in the evaluation of health impact assessments. Public Health.

[57] Craig P., Dieppe P., Macintyre S., Michie S., Nazareth I., Petticrew M. (2008). Developing and evaluating complex interventions: The new Medical Research Council guidance. BMJ.

[58] Hawe P., Bond L., Butler H. (2009). Knowledge theories can inform evaluation practice: What can a complexity lens add?. New Dir. Eval..

[59] Stame N. (2004). Theory-Based Evaluation and Types of Complexity. Evaluation.

[60] Rogers P.J. (2008). Using Programme Theory to Evaluate Complicated and Complex Aspects of Interventions. Evaluation.

[61] Jabot F., Tremblay E., Rivadeneyra A., Diallo T.A., Lapointe G. (2020). A Comparative Analysis of Health Impact Assessment Implementation Models in the Regions of Montérégie (Québec, Canada) and Nouvelle-Aquitaine (France). Int. J. Environ. Res. Public Health.

[62] Buregeya J.M., Loignon C., Brousselle A. (2020). Contribution analysis to analyze the effects of the health impact assessment at the local level: A case of urban revitalization. Eval. Program Plann..

[63] Brousselle A., Buregeya J.-M. (2018). Theory-based evaluations: Framing the existence of a new theory in evaluation and the rise of the 5th generation. Evaluation.

[64] Høydal Ø.S. (2018). Evaluation: Method and societal phenomenon. Evaluation.

